# The Journey of Saudi Male Nurses Studying within the Nursing Profession: A Qualitative Study

**DOI:** 10.3390/nursrep11040078

**Published:** 2021-10-25

**Authors:** Maram Banakhar, Maha Bamohrez, Raghad Alhaddad, Reema Youldash, Rwan Alyafee, Sufanah Sabr, Loujain Sharif, Alaa Mahsoon, Nofaa Alasmee

**Affiliations:** 1 Public Health Nursing Department, Faculty of Nursing, King Abdulaziz University, Jeddah 21589, Saudi Arabia; 2 Faculty of Nursing, King Abdulaziz University, Jeddah 21589, Saudi Arabia; raghad.alhaddadd@outlook.com (R.A.); reemayouldash@gmail.com (R.Y.); ra.iii@hotmail.com (R.A.); sufanahsabr@gmail.com (S.S.); 3 Psychiatric and Mental Health Nursing Department, Faculty of Nursing, King Abdulaziz University, Jeddah 21589, Saudi Arabia; lsharif@kau.edu.sa (L.S.); mahsoon@kau.edu.sa (A.M.); nalasmee@kau.edu.sa (N.A.)

**Keywords:** male nurses, gender bias, social media

## Abstract

Background: Nursing is considered to be a primarily female profession, particularly in Saudi Arabia, despite the fact that male nurses have contributed to the advancement of the nursing profession in various specialties, such as military nursing, mental health, and critical care. Purpose: We explore the factors influencing Saudi male nursing interns to study within the nursing profession in Saudi Arabia. Methods: A descriptive qualitative study was conducted. A convenience sample of 12 Saudi male nursing interns from different nursing colleges were recruited, alongside four hospital internship coordinators. The data were collected by conducting two semi-structured focus group interviews and four individual interviews. All the interviews were analyzed using a comparative analytical approach. Results: Role models and the role of the internship year were identified as factors influencing Saudi male nurses’ decision to study nursing. However, hospital placements, cultural preferences, and the preferences of patients and their families for female nurses were the key challenges encountered. Importantly, this study demonstrated that social media plays a critical role in raising awareness regarding the importance of Saudi male nurses. **Conclusion**: Awareness needs to be raised of the nursing profession as a gender-diverse field through volunteering programs for the community. It is recommended that Saudi male nurses act as role models for students in the Academic Orientation Forum and on social media.

## 1. Introduction

The nursing literature has highlighted several factors influencing male nurses’ decisions to enter the nursing profession. For instance, a mixed methods review reported the challenges faced by males considering entering the nursing profession. These involve social and educational challenges, career choice, recruitment, and the pressures of the role [1]. Moreover, a further cross-sectional study carried out in Taiwan by distributing questionnaires to 148 nursing students showed that male nursing students face a high to medium amount of stress in achieving their life goals and in their learning, which is higher than the interpersonal life stress they experience [2].

In addition to the above, various studies have examined attitudes towards male nurses. One quantitative study carried out in Ghana explored the preferences of female patients receiving nursing care from male nurses. It was found that female patients described male nurses as courteous and reported that their treatment was comfortable, which indicated a positive attitude [3]. A similar result was obtained in a descriptive study that distributed questionnaires to mothers of hospitalized children receiving treatment in Turkey. The results showed that respondents considered nursing as not being solely for females (55.5%) and were of the opinion that male nurses have a role in healthcare settings (85.5%). Importantly, the results indicated that male nurses should be available to deal with male patients (33.6%), and gender should not be discriminated against in the nursing profession (20%) [4]. In contrast to previous studies, another study conducted in Jamaica assessed patients’ attitudes towards male nurses by distributing an attitudinal measuring scale to 80 patients who received care from both female and male nurses. It was found that only 10% had a negative perception of the care received from male nurses. Moreover, 80% of male and 54% of female patients reported a negative attitude towards the male nurses who gave them an enema [5].

Furthermore, two qualitative studies explored the perception of male nursing students in providing care. In Oman, a focus group interview study showed that there are several barriers that restrict the care given to children, such as communication, socio-cultural issues, and a lack of support from the health team, faculty, and society. In addition, male nurses face gender bias, as well as barriers such as low self-esteem, which can make it more difficult to provide care for children and may reduce male nurses’ confidence levels [6]. A further qualitative study in Hong Kong that examined therapeutic relationships within private and public hospitals revealed that the presence of a relative, the social context, the type of procedure, and the attitudes of female patients are significant in the development of such relationships [7]. Another study aimed to compare the perceptions of undergraduate male nursing students regarding the image of the nursing profession in Jordan and Egypt using questionnaires. This demonstrated that there is a significant positive perception of the nursing profession among male nursing students in relation to descriptions of the nursing profession, societal views, the benefits of the nursing profession, and levels of self-satisfaction in nursing [8].

The challenges faced by male nurses in the nursing profession have been extensively examined in the nursing literature. For instance, gender bias was reported to be the most common challenge faced by male nurses in various studies. One study aimed to identify the experiences of male nursing students in the nursing profession by conducting two focus group interviews with third- and fourth-year students. The findings indicated that nursing instructors could be more enthusiastic in promoting a convenient learning setting for male nursing students, and should highlight the history of male nurses in their classes to inspire male students [9]. Similarly, another study conducted interviews with third- and fourth-year male nursing students at two universities in Turkey. The results showed that male nursing students do not gain sufficient knowledge and skills about services, including care before and after delivery and family planning, which are categorized as primary health services. They are also not given the opportunity to put their knowledge into practice [10].

Furthermore, patients refusing care from male nurses, the lack of male nurses to act as role models, as well as the negative effects of cultural stereotypes on male nursing students were all reported as challenges after conducting individual interviews with 11 male nursing students [11]. Moreover, in another study, four focus group interviews (*n* = 20) showed that male nursing students encountered gender discrimination in both college admission policies and hospital policies that favor females over males. Additionally, negative stereotypes from students of other academic majors, and negative self-image, were also encountered by male nursing students [12].

A literature review identified that while each society has a different culture, they all agree with the idea of men entering nursing, although those who do face many challenges. One of the most difficult widely reported challenges is the idea of the feminine touch and intimate examinations. Simultaneously, people hold different views on male doctors and male nurses, as most people do not mind if there is a male doctor in the room [13]. Additionally, a further study emphasized that female nurses were less accepting than their male counterparts. A study carried out in the USA that aimed to identify attitudes towards male nurses examined correlations between female nurses’ levels of acceptance and certain demographic variables by distributing an online survey to 60 male and 251 female nurses from three different medical centers revealed that the accepting attitude of male nurses was higher than that among female nurses [14].

Furthermore, a mixed-methods study conducted in the USA used surveys and interviews to examine the challenges faced by non-traditional male students. It was found that most participants found it difficult to achieve a balance among family, school, and work, with a gender bias being the most challenging aspect they faced [15]. Moreover, with regard to the readiness of male nurses to practice as Registered Nurses (RNs), focus group interviews (*n* = 22) highlighted negative attitudes towards nursing, a lack of family support, mistrust by patients, gender discrimination, a lack of interpersonal communication, and a failure to include male nursing students in professional discussions [16]. In addition, a literature review found that nursing remains a female-dominated career, and men who are interested in entering this profession are questioned about their sexual orientation and considered inappropriate as bedside nurses [17]. A quantitative study that compared female and male nursing students in terms of self-reported competence found that female students regard themselves as competent at providing direct nursing care, while male students regarded themselves as more competent in management and leadership [18].

In 1958, the first one-year program for males in the nursing profession in Saudi Arabia was established in Riyadh under the administration of the Ministry of Health (MOH) in collaboration with the World Health Organization (WHO) [19]. In 2004, male nursing Bachelor level programs were established, such as at King Khalid University and King Saud University. Recently, according to the Ministry of Health, more than 10,000 male nurses now hold a diploma degree [20]. The nursing profession in Saudi Arabia has improved and there are now upgraded Bachelor level programs and postgraduate degrees for male nurses. Saudi Arabia has a wealth of information from the international nursing field that can be used to improve its own healthcare system and manage its human resources to meet the needs of the 21st century [21].

These issues and challenges encountered by male nurses in terms of entering and studying nursing as a profession reported in the nursing literature have had major consequences, such as embarrassment and guilt when taking care of female patients [7]. Furthermore, in the context of Saudi Arabia, although there is an urgent need for Saudi male nurses to work in several healthcare organizations and achieve the goals set out in Kingdom Vision 2030, there are very few nursing programs for male students. Importantly, the majority of Saudi male nurses experience low levels of confidence and low self-esteem due to social norms and cultural stereotypes [7]. Moreover, it is very important to note that Saudi male nurses’ duties are limited to male care only, and male nurses are not allowed to provide nursing care to pediatric, obstetrics, and gynecology patients [7,11]. Additionally, to the best of the researchers’ knowledge, no study has yet been carried out to explore the experiences of and challenges faced by Saudi male nurses in studying nursing at any nursing program. Therefore, this study provides in-depth information regarding the experiences of Saudi male nurses in entering and studying within the nursing profession and the challenges faced, as well as justifications given for the shortage of Saudi male nurses in Saudi Arabia. The findings of this study will be beneficial for nursing colleges, institutions, and healthcare organizations and should help to improve the image of Saudi male nurses in Saudi Arabia. This study, therefore, aimed to explore the factors influencing Saudi male nursing interns to study within the nursing profession in Saudi Arabia. The following research questions were addressed: How do Saudi male nurses experience studying nursing in Saudi Arabia? What challenges do Saudi male nurses face when studying nursing in Saudi Arabia?

## 2. Materials and Methods

### 2.1. Research Design

**Figure 1:**
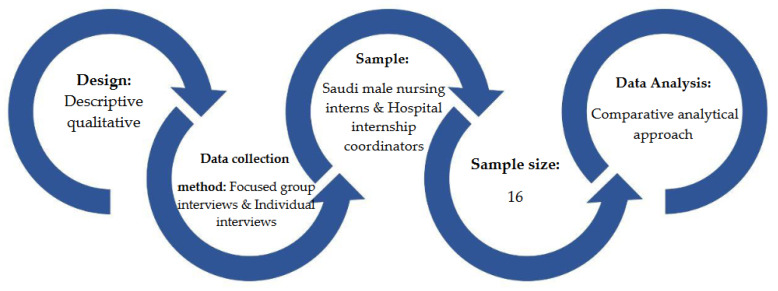
Study methods.

A descriptive qualitative research design was developed to provide in-depth information regarding the experience of Saudi male nurses in the nursing profession, as qualitative research tends to be holistic, aiming to achieve an understanding of the field as a whole [22], see Figure 1.

### 2.2. Study Setting and Participants

A convenience sampling technique was used to select the study participants. Convenience sampling uses participants who are accessible, available, and willing to participate [23]. The sample in this study consisted of two groups of participants: Saudi male nursing interns and nursing internship coordinators. In this context, Saudi male nursing interns were drawn from different Bachelor level nursing programs to explore their experiences and challenges in studying and entering the nursing profession in Saudi Arabia. Furthermore, internship nursing coordinators were also recruited from different hospitals to explore their experience in dealing with Saudi male nurses and the challenges they faced during their hospital placements. The Saudi nursing interns were recruited from one private and two governmental nursing colleges in the western region of Saudi Arabia. These nursing colleges were selected as they provide Bachelor level nursing programs for Saudi male nurses and were accessible for the researchers. The nursing internship coordinators were recruited from four different hospitals (two Ministry of Health (MOH) hospitals, one specialized hospital, and one teaching hospital).

The inclusion criteria incorporated any Saudi male nursing intern in the internship year of a nursing program at any private or governmental nursing college. Male nursing students in their first, second, or third year of study were excluded as they are students studying at the college and, therefore, cannot provide answers to the research questions; however, students currently in the internship year, which is the final year in the nursing program, can describe and explain their experience in more detail after finishing all years of study. For nursing internship coordinators, the inclusion criteria were any Saudi or non-Saudi nursing coordinators in the hospital internship program with Saudi male nursing interns. 

Considering the sample size in the current study, the planned sample size ranged between 16 and 20 participants. However, the number of recruited participants was 22 (17 male nursing interns and 5 nursing internship coordinators), while the sample size in this study was 16 participants (12 Saudi male nursing interns and 4 nursing internship coordinators) based on data saturation, as no new information emerged from either of the focus group interviews or individual interviews. In this context, the information and experience obtained from the Saudi male nursing interns were repeated in the second focus group and in the fourth individual interview with the internship nursing coordinators. Therefore, the authors decided to stop the data collection, as they found they reached data saturation. Saturation can be achieved in a small sample if participants communicate effectively [23].

Ethical approval for the current study was obtained from the faculty of nursing in one academic institution where the site of the study originates (NREC Serial No: Ref No 1B. 91). After obtaining ethical approval, the study participants were recruited online through social media (Twitter) due to the fact that the study was conducted at the start of the COVID-19 pandemic crisis and there were quarantine requirements in Saudi Arabia; therefore, other institutions were unable to accept any approval requests during the pandemic crisis. Hence, a social media platform (Twitter) was used to contact both groups of participants and invite them to participate in the study. In this context, a Google form was created to recruit the participants. In this study, the data collection period ran from March to May 2020. Our manuscript complies with COREQ reporting guidelines.

### 2.3. Data Collection Method

The data were collected by conducting both semi-structured focus groups and individual interviews. Two semi-structured focus group interviews were conducted via Zoom for Saudi male nursing interns to provide broad and in-depth information, clarify participant details, and confirm insights [23]. Each focus group involved six participants from different programs who had different experiences and opinions. In the focus group interviews, the moderator guided the discussion using a prepared topic guide to ask the participants questions. The interview guide for this study was designed after reviewing the literature with the objective of exploring Saudi male nursing interns’ experiences of studying nursing in Saudi Arabia, the factors that influence Saudi male nurses’ decisions to study nursing, and to explore the challenges they face from multiple perspectives. All questions were reviewed and updated after each focus group interview. All focus group interviews were recorded and lasted for one hour.

Moreover, four semi-structured individual interviews were carried out with nursing internship coordinators via Zoom to explore their individual experiences of the challenges faced in dealing with Saudi nursing interns in the internship year, as well as the differences between male and female nursing interns. The interview questions were developed after reviewing the literature and included the following: the need for Saudi male nurses and their placements, challenges in clinical training, and how Saudi culture influences Saudi male nursing interns. All questions were reviewed and updated after each interview. All individual interviews were recorded and lasted between 30 min and one hour.

### 2.4. Data Analysis

The data obtained from the individual interviews and focus groups with different groups of participants in this study were analyzed using the comparative analytical approach in order to develop codes, categories, and themes by coding and analyzing the data [24]. Therefore, three stages were used in this study to compare and contrast the collected data. All conducted interviews were transcribed verbatim. The first stage involved a comparison with each individual and focus group interview to develop the initial codes. In this context, each transcript was read carefully two times to identify relevant and important text using a line-by-line mode of analysis. All identified initial codes were labeled, and these labels involved the participants’ actual words and descriptions. Furthermore, each initial code identified in each transcript was labeled after three to four times. In the second stage, the data analyzed from the individual interviews were compared to note any similarities or differences within the initial codes and to develop the categories. In addition, the same was carried out for the data analyzed from the focus group interviews. Comparing data from different interviews in this way was important, as dealing with the same category enabled the same codes to be assigned to similar types of data. The last stage involved a comparison between both groups of participants. In this context, the initial codes and categories generated from individual interviews were compared and contrasted with the codes and categories obtained from the focus group interviews to generate the final themes.

### 2.5. Ethical Considerations

An informed consent form was signed by each participant. The consent form clearly stated that the participant had the right to refuse to participate in the study at any time without giving a reason, and that the interviews would be recorded. In addition, the participant information sheet for each group of participants provided all necessary information about the study, such as the aim, reasons for selecting each group of participants, and the voluntary nature of participation in the study. Moreover, participants’ names were removed, and each participant was assigned a code during the process of data analysis and reporting the findings of the study. The confidentiality of study participants by using the Zoom application was maintained by using the waiting room function, which locks the interview meeting and requires a password to enter. Importantly, there was only one host controlling the interview meeting and allowing the entrance of study participants.

## 3. Results

A total of 16 study participants participated in this study with different sample characteristics, as demonstrated in Table 1.

**Figure 2:**
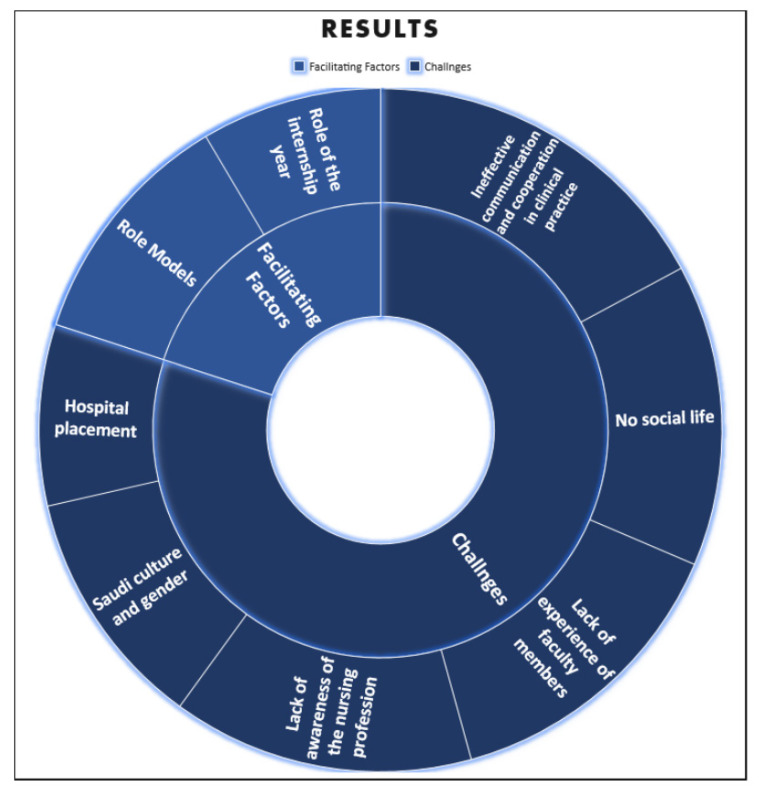
Study themes and sub-themes.

Two major themes emerged from the conducted interviews. These involved facilitating factors and the challenges faced in studying nursing in Saudi Arabia, as shown in Figure 2 and Table 2 below.

### 3.1. Facilitating Factors

#### 3.1.1. Role Models

In this study, the analysis showed that some of the male nursing interns opted to study nursing based on the advice of others, as there are considered to be good opportunities for Saudi males to be hired quickly by hospitals: “I got into nursing based on people’s advice, and since it’s a new major in our university, this is a new opportunity and I wanted to give it a try. At first, I was majoring in engineering, but I switched to nursing.” (Nurse 7 “Nr 7”). Additionally, having a role model (a nurse) in the family was helpful in encouraging participants to select nursing as a profession, as well as raising awareness of what the nursing profession entails: “My uncle has been a registered nurse for 25 years, so I almost had a background in nursing as a profession, the nature of the work, the future of this profession and the job description of it. That’s why I chose nursing.” (Nr 8).

#### 3.1.2. Role of the Internship Year

The majority of Saudi male interns considered the internship year to have a crucial role, as it helped them to understand the nursing profession in the real world, including roles and responsibilities: “Every clinical experience had an influence on me, even the internship year. I understood what the nursing profession means. We understood the things we used to hear in the theory classes, and how different it is in real life.” (Nr 8). Moreover, the internship year was very helpful for all Saudi male nursing interns in terms of increasing their level of knowledge of various clinical issues and topics: “One of the advantages of nursing is that it enables me to discuss and share ideas on several topics in various areas.” (Nr 2).

Additionally, Saudi male nursing interns pointed out that the exposure gained during the internship year helped them to understand how to deal with different types of patients, as well as the emotional feelings of each patient and their family: “Each nurse is allocated to five or six patients and I have to know how to deal with each patient and their families’ emotional feelings.” (Nr 11). “A positive thing is that I learned how to deal with a mentally ill patient or a patient who’s in so much pain.” (Nr5). Similarly, being sufficiently patient to understand and deal with different cultures and communicate with non-Saudi nurses, as well as showing commitment in the workplace, were further important results of the internship year mentioned by male nursing interns: “Nursing made me more committed in my life” (Nr 11). “Patience, that’s the most important lesson I’ve learned, and how to deal and communicate with different cultures and with non-Saudi nurses.” (Nr 3). However, it was noted that Saudi male nursing interns had only recently begun to be accepted into hospital internship programs due to the need for Saudi male nurses to be recruited by hospitals. This was confirmed by all internship coordinators at different hospitals. “In previous years the internship program in our hospital was limited to female nursing students, however, last year we started to accept two to three male nursing interns because we will need them in our hospital as staff in the future.” (Internship Nursing Coordinator “INC 2”); “We need Saudi male nurses in our hospital because of the severe shortage that we have, therefore, it is important and must to accept in our internship program Saudi male nursing interns every year” (INC 4).

### 3.2. Challenges

#### 3.2.1. Lack of Awareness of the Nursing Profession

The lack of awareness of nursing as a profession was reported to be a challenge encountered by Saudi male nursing interns in the current study. It was found that the majority were unaware of nursing and what constitutes the nursing profession when they first enrolled at university, which resulted in them changing their major from nursing to another specialty: “I mean we are going by the image that society creates (injections and vital signs) and that’s it. So, half of the batch had the idea of dropping out in the first or even second semester.” (Nr 8).

A common view amongst interviewees was that families were unsupportive due to a lack of awareness regarding the nursing profession. For example, the majority of participants stated that nursing is considered to be limited to taking vital signs and giving injections: “My family were refusing the idea of me majoring in nursing, and to be honest being a nurse wasn’t my ambition; I wanted something else, because my family and I had the idea that nursing is only about injections.” (Nr 10). Furthermore, a lack of awareness in the community regarding the nursing profession was also reported. In this context, it was felt that social media is crucial in raising the level of awareness in the community. This was described as follows by one Saudi male nursing intern who volunteered in community campaigns: “Volunteering has a significant role in raising awareness. Honestly the thing that helped me and my colleagues was that we were able to explain about male nurses’ role in the community. People in our society they are not used to the idea of Saudi male nurses as they are more used to females.” (Nr 8).

Consequently, the findings showed that all participants lacked confidence when they entered the nursing profession due to a lack of awareness of the nursing profession, in particular, when they started to study new terminology and subjects at college: “The first year was difficult due to the new terminology in anatomy and physiology subjects, but in the second year we began to understand medical terms more.” (Nr 9). Nevertheless, after graduating from nursing college and becoming more informed about the profession, they felt more confident about being a Saudi male nurse and working within the community: “After I studied nursing I became more informed about the nursing profession; now I can face anyone who reduces the importance of nursing.” (Nr 10).

#### 3.2.2. Lack of Experience of Faculty Members

One of the significant challenges faced by Saudi male nursing interns was the lack of experience of faculty members. The interviews highlighted that instead of having faculty members specializing in nursing in the male section of the college, male faculty members from another specialty were recruited to teach all nursing subjects, which resulted in a lack of interest in attending or understanding the subject: “For example, the nursing course was taught by a lecturer who specializes in Microbiology, and he had no idea how to explain the procedure; it was like a student was reading it.” (Nr 2).

Moreover, having a faculty member who is not from the nursing specialty, in particular, in maternity and pediatric specialties, was mentioned as a challenge faced by Saudi male nursing interns. In this context, participants revealed that they did not gain any clinical experience at university in either of the courses due to staff shortages; a lack of faculty members’ experience in using simulations; and ineffective teaching methods, such as using pictures: “During the first year, we used to encounter some issues and difficulties regarding the simulation labs, although they were provided with the necessary equipment.” (Nr 8). “Although labs were available for these two subjects, we didn’t practice the procedures because the doctor was a little shy, so he was using pictures instead of an application; even the OSCE exam was through presenting pictures.” (Nr 11).

#### 3.2.3. Hospital Placement

The hospital placement for clinical training during the courses was a crucial challenge faced by all the participants. In this context, Saudi male nursing interns encountered difficulties in finding a hospital placement for their clinical training due to the priority being given to female nursing students in crowded hospital units: “At the beginning we suffered, especially in the training, because not all the hospitals were open, and not all of them wanted male nursing students in comparison to female students because of their number; they were 105 students, and we were only 25 students.” (Nr 8).

In terms of clinical training, it was found that participants did not learn or interact with patients during their courses because the main focus was on paperwork: “I feel like I didn’t get the benefit from the clinical training. The reason was they always ask us for paperwork, like cases and nursing care plans, flow sheets and things like this, there isn’t enough time for you to fill in the paper.” (Nr 1).

The lack of a faculty member to act as a clinical instructor for male nursing students was found to be a reason why male students are not accepted for training. This was emphasized by nursing internship coordinators, who stated that there is limited capacity for Saudi male nurses to receive hospital training during their courses in comparison to Saudi female nursing students, while in other hospitals, male nursing students are not accepted for training. “We don’t accept male nursing students for training within the program because there are no clinical instructors from the college who would have a similar role to the instructor for the female nursing students, which is against the training unit protocols.” (INC 1). “For us in this hospital we can accept only limited numbers.” (INC 4).

#### 3.2.4. Saudi Culture and Gender

Importantly, in clinical settings, the cultural issues faced by participants who were not able to provide care to female patients according to the faith and religious beliefs of the community or certain religions were cited as a significant challenge for particular nursing procedures. Furthermore, it was thought that the cultural issue might be more challenging in some cities than others, such as Mecca and Taif: “We had some ethical issues concerning maternity, so we had to just study the theoretical part, we only cared about getting the grade and passing the course without understanding the content”; “It is a cultural issue; it would be stricter in Taif and Mecca than in Jeddah” (Nr 6).

A further challenge was the preference of some patients or their families for female nurses due to their cultural beliefs: “During my duties, I have had several cases with a female patient or her family who strictly stick to their culture, and refuse me and my medical services. They used to ask for female nurses only.” (Nr 7).

Female nurses’ commitment as well as their availability during shifts were the main reasons for preferring female over male nursing interns. This was confirmed by all nursing internship coordinators as follows: “The main challenge of having male nursing interns is that they come late, showing no commitment as well as leaving early their duties.” (INC 3). Moreover, cultural issues mean that the male interns are not allowed to practice clinical rotations in obstetrics/gynecology wards and pediatric wards: “Their Rota doesn’t include maternity and pediatric rotations because of Saudi culture as you know.” (INC 1).

#### 3.2.5. No Social Life

Likewise, one of the major challenges on which all participants agreed was the 12-h shifts. In this context, the participants expressed concerns about their social life while working 12-h shifts and how they could plan and manage their own life: “In the future it would be very tiring to work for 12 h, for example when I get married and have a family, no way I would accept a 12-h shift.” (Nr 7).” Working for 12-h shifts will let you to feel that you are lonely, no socialization and no life” (Nr 2).

#### 3.2.6. Ineffective Communication and Cooperation in Clinical Practice

In this context, a language barrier was experienced by the majority of male nursing interns when they requested particular information or asked a question. One interviewee noted:

“Most of the staff are from foreign countries, so the only challenge was in communication and how to ask for certain information or a question.” (Nr 10).

Moreover, as there are staff of different nationalities and expatriate nurses in the hospitals, female nurses were uncooperative with Saudi male nurses in clinical practice, refusing to help them on the pretext that they were busy and had no time: “Some nationalities weren’t very helpful. Filipinos though are very helpful, but Indians not so much; they are not cooperative, only a few of them are helpful.” (Nr 7).

However, the internship coordinators highlighted that the language barrier is a common issue for Saudi male nursing interns. Their lack of proficiency in English resulted in ineffective communication between Saudi male interns and foreign staff. “Difficulty in speaking and communicating in English is a common problem we face with Saudi male interns every year and this is because of their university; when they have their own courses they don’t have them in English.” (INC 1).

On the other hand, all interns, whether male or female, must be able to speak and communicate in English as a requirement for acceptance as a hospital intern and as a member of staff at the end of their internship program. This was described by one internship coordinator: “Any nursing student who is interested in applying for their internship year or as a member of staff in our hospital must first pass a written English exam because English is the language spoken in the hospital with people of different nationalities.” (INC 1). 

## 4. Discussion

Although there is a need to hire Saudi male nurses in different healthcare organizations, particularly with the COVID-19 pandemic crisis, there are several challenges faced by male nurses in studying nursing as a profession in Saudi Arabia. The current study revealed that having a role model is a facilitating factor and plays a key role in selecting nursing as a profession in Saudi Arabia. This was similar to another study conducted in the United States, which found that having role models of male nurses is crucial [11]. This result can be explained by the fact that having a role model of a Saudi male nurse can help to increase the level of awareness and provide a positive image regarding the profession, as well as the opportunities that are exclusive to nursing. 

The most important relevant finding in this study was the lack of awareness regarding the nursing profession. This finding is consistent with another study in Saudi Arabia, which demonstrated that most of the participants’ families, relatives, and friends, and the public perceived the nursing profession to be an unsuitable career for males [16]. This finding may be due to the limited number of nursing colleges that provide nursing programs for Saudi male nurses, a lack of understanding regarding the crucial need for Saudi male nurses in hospitals, as well as the limited availability of role models to inspire and raise awareness of the nursing profession for Saudi males. Thus, it is important to raise awareness of the need for Saudi male nurses using different methods in nursing colleges in order to increase the number of nurses and address this shortage. Importantly, this study indicated that social media plays a key role in increasing community awareness regarding the need for Saudi male nurses. In accordance with the present findings, a previous study suggested that social media could be used as a medium for raising awareness and knowledge of nursing issues [25]. Similarly, another study reported that social media is an important tool that enables nurses to interact with other people and quickly reach international audiences [26]. A possible explanation for this is that social media is easy to use and accessible by different generations. Additionally, it has been found to be the fastest and most effective way of spreading information. Hence, being active on social media is an important aspect of representing a suitable image of nursing as a profession and to increase the level of awareness in the community.

Another important finding of the current study was the difficulty of being accepted onto a hospital placement for clinical training during nursing courses, as reported by both groups of participants. This finding is similar to that of a study that confirmed that male nursing students face gender discrimination when it comes to acceptance for clinical training at hospitals [12]. This result might be explained by the fact that hospitals are overcrowded, with a large number of female nursing students, specifically, in two to four governmental and private universities, all being based in one city. Moreover, hospital coordinators prefer female to male students for several reasons, such as their commitment and availability of clinical instructors. Hence, defining the capacity or number of students to be accepted into each hospital for both male and female nursing students is important in order to provide a fair opportunity for both genders for clinical training [27,28].

In this study, both Saudi male nursing interns and nursing internship coordinators revealed that the exposure gained during the internship year plays a key role in increasing the level of knowledge and awareness of the nursing profession and confidence in it. This finding is explained by the opportunities provided in the internship year to experience a realistic working environment as a staff nurse in different healthcare organizations and to gain an insight into the practice of professional nursing. Several reports have shown that an internship year led to a noticeable increase in the confidence levels of students, as well as strengthening their self-esteem, knowledge, and understanding and better equipping them for their jobs. Therefore, providing a comprehensive internship program in the internship year is crucial to prepare students for life as a member of staff in the future [29,30].

Similarly, it was found that cultural issues faced by Saudi male nurses are a common challenge, leading to a lack of clinical experience in two different specialties, namely, maternity and pediatrics, which is particularly found in Saudi Arabia, as reported by both groups of participants. This finding seems to be consistent with other studies, which found that male nurses handled cultural challenges, such as the therapeutic feminine touch, from a different perspective to that understood by the society in which they live [13]. These results are likely to be related to the social norms in Saudi Arabia as well as cultural barriers, e.g., the gender difference means that males and females in Saudi Arabia cannot intermingle and interact in public places or in hospital units [31]. In accordance with the results of the current study, previous studies have indicated that cultural and ethical issues in Arab countries have prevented male nurses from gaining clinical experience in hospitals, which affects their ability to manage maternity patients [32]. Moreover, as a result of the culture in Saudi Arabia, male Saudi nurses graduate from the program without having practiced maternity or pediatric clinical rotations in their courses or in their internship year and, therefore, the nursing program is incomplete, whereas Saudi male medical students are allowed to practice the two specialties within the program as well as in their internship year without any barrier or obstacle [33]. However, this is inconsistent with international nursing programs, which allow male nursing students to practice maternity and pediatrics clinical rotations in the same way as female nursing students without any discrimination or cultural issues [34].

This study indicated that patients and their relatives prefer female to male Saudi nurses. However, this finding is contrary to a previous study conducted in Ghana, which found that patients prefer male nurses to female nurses. Female patients were comfortable with having their care provided by a male nurse, and described them as polite [3]. There are several possible reasons for this result, one of which is that nursing is perceived as a female profession. Culture also has an effect, as Saudi male nurses are subject to stereotyping and misconceptions. Therefore, raising public awareness about the nursing profession and, in particular, the role of Saudi male nurses is highly recommended. In the current study, working 12-h shifts was highlighted as a challenge by the study participants, as it affected their social lives. In accordance with the present results, a previous study found that nurses who work 12-h shifts experienced excessive fatigue, back pain, and sleeplessness for over a week. As a result, their work consumed most of their time and effort and diminished their chances of maintaining an active social life [16]. A possible explanation for this result may be the fact that working 12-h shifts is considered by healthcare organizations to be a long time spent at work and causes fatigue. However, it is important to note that 12-h shifts are required at specialized hospitals, teaching hospitals, and military hospitals, while nurses at Ministry of Health hospitals work 8-h shifts.

A further challenge highlighted in this study by both groups of participants was the ineffective communication between Saudi male nurses and female foreign nurses. The findings of the current study are consistent with those from a previous study, which showed that male nurses considered interpersonal communication to be a major challenge [16]. There are two possible explanations for this result. First, the ineffective use of English at colleges plays a key role. Second, the lack of exposure during clinical training in the hospital affects the use of English by Saudi male nurses. Hence, increasing the use of English in university lectures for all students and for communication, as well as spending long hours in clinical training, are important [35].

In terms of the limitations, this study was affected by the COVID-19 situation, as the data were collected from March 2020, which affected the researchers’ work in many ways, such as the ability to use a large sample of participants from different geographical areas, as well as technical issues with the Internet. Moreover, the different schedules, duties, and responsibilities of the recruited participants also affected their ability to participate in the focus group interviews to conduct the study.

The trustworthiness of the study was ensured through two different methods. First, triangulation of data collection methods was achieved by conducting both individual and focus group interviews. A further use of triangulation incorporated two groups of participants, namely, Saudi male nursing interns and nursing internship coordinators. Each group helped the researchers to answer the research questions by comparing the similarities and differences between the generated ideas and experiences. Moreover, recording the interviews and transcribing them verbatim were additional strategies employed to minimize any systematic bias and ensure the reliability of the collected data [36,37]. Transcribing the interviews verbatim has the advantage of providing the opportunity for all researchers to become fully immersed in the data.

## 5. Conclusions

This study aimed to explore the factors influencing Saudi male nursing interns’ decisions to study within the nursing profession in Saudi Arabia. The current study showed that a lack of awareness of nursing as a career option for males and the role of Saudi culture were identified as challenges experienced by Saudi male nurses in studying nursing. Moreover, social media plays a critical role in raising awareness of the importance of Saudi male nurses. Hence, it is important to raise awareness of the nursing profession through volunteering programs for the community, as well as explaining the importance of the nursing profession for students in the Academic Orientation Forum and on social media. Furthermore, it is recommended to present different Saudi male nurse role models who can inspire students during their university education years. The use of simulation scenarios within courses or in the internship year to replace clinical training in maternity and pediatric courses, in addition to allowing Saudi male nursing students to practice in the hospital, is also suggested. Importantly, there is a definite need for hospital administration to allow Saudi male nurses to practice clinical rotations and practice in both maternity and pediatric units in the same way as male physicians. Future research into the level of knowledge of, as well as perceptions and attitudes towards, Saudi male nurses among both healthcare workers and the public is highly recommended.

## Figures and Tables

**Table 1:** Characteristics of study participants.

Sample Characteristics	Frequency (%)
**Occupation**	
▪ Saudi male nursing interns ▪ Internship nursing coordinators	12 (75%) 4 (25%)
**Gender**	
▪ Male ▪ Female	12 (75%) 4 (25%)
**Age**	
▪ <23 years old ▪ 24–34 years old ▪ 35–45 years old ▪ >45 years old	12 (75%) 1 (6.25%) 2 (12.5%) 1 (6.25%)
**Institution**	
▪ Governmental institution ▪ Private institution	14 (87.5%) 2 (12.5%)

**Table 2:** Themes, sub-themes, and number of participants.

Theme	Category	No. of Participants	Similar\Different from the Literature
**Facilitating factors**	Role models	6 Saudi male nursing interns	Similar theme to the literature
	Role of internship	16 Saudi male nursing interns and nursing internship coordinators	New theme emerged from the data
**Challenging factors**	Lack of awareness of the nursing profession	12 Saudi male nursing interns	New theme emerged from the data
	Lack of experience of faculty members	10 Saudi male nursing interns	New theme emerged from the data
	Hospital placement	10 Saudi male nursing interns 4 nursing internship coordinators	New theme emerged from the data
	Saudi culture and gender	16 male Saudi nursing interns and nursing internship coordinators	Similar theme to the literature
	No social life	8 Saudi male nursing interns	New theme emerged from the data
	Ineffective communication and cooperation in clinical practice	9 Saudi male nursing interns 4 nursing internship coordinators	New theme emerged from the data

## Data Availability

Not applicable.
